# Building the capacity of users and producers of evidence in health policy and systems research for better control of endemic diseases in Nigeria: a situational analysis

**DOI:** 10.1186/s12992-019-0530-6

**Published:** 2019-11-21

**Authors:** Obinna Onwujekwe, Enyi Etiaba, Chinyere Mbachu, Uchenna Ezenwaka, Ifeanyi Chikezie, Ifeyinwa Arize, Chikezie Nwankwor, Benjamin Uzochukwu

**Affiliations:** 1 Health Policy Research Group, Department of Pharmacology and Therapeutics, College of Medicine, University of Nigeria Enugu-Campus, Enugu, Nigeria; 2 Department of Health Administration and Management, Faculty of Health Science and Technology, College of Medicine, University of Nigeria Enugu-Campus, Enugu, Nigeria; 3 Institute of Public Health, College of Medicine, University of Nigeria Enugu-Campus, Enugu, Nigeria; 4 Department of Community Medicine, College of Medicine, University of Nigeria Enugu-Campus, Enugu, Nigeria

**Keywords:** Capacity building, Endemic diseases, Health policy and systems research+ analysis, Producers of evidence, Users of evidence

## Abstract

**Background:**

There is a current need to build the capacity of Health Policy and Systems Research + Analysis (HPSR+A) in low and middle-income countries (LMICs) as this enhances the processes of decision-making at all levels of the health system. This paper provides information on the HPSR+A knowledge and practice among producers and users of evidence in priority setting for HPSR+A regarding control of endemic diseases in two states in Nigeria. It also highlights the HPSR+A capacity building needs and interventions that will lead to increased HPSR+A and use for actual policy and decision making by the government and other policy actors.

**Methods:**

Data was collected from 96 purposively selected respondents who are either researchers/ academia (producers of evidence) and policy/decision-makers, programme/project managers (users of evidence) in Enugu and Anambra states, southeast Nigeria. A pre-tested questionnaire was the data collection tool. Analysis was by univariate and bivariate analyses.

**Results:**

The knowledge on HPSR+A was moderate and many respondents understood the importance of evidence-based decision making. Majority of researcher stated their preferred channel of dissemination of research finding to be journal publication. The mean percentage of using HPSR evidence for programme design & implementation of endemic disease among users of evidence was poor (18.8%) in both states. There is a high level of awareness of the use of evidence to inform policy across the two states and some of the respondents have used some evidence in their work.

**Conclusion:**

The high level of awareness of the use of HPSR+A evidence for decision making did not translate to the significant actual use of evidence for policy making. The major reasons bordered on lack of autonomy in decision making. Hence, the existing yawning gap in use of evidence has to be bridged for a strengthening of the health system with evidence.

## Background

Endemic tropical diseases continue to impose a tremendous health burden in resource-poor countries throughout the world, claiming millions of lives annually and inflicting severe morbidity that results in significant losses in economic productivity and social progress [1]. Nigeria did not meet the Millennium Development Goals (MDG) targets for malaria which was to halt and reverse the spread of malaria and some other communicable diseases such as HIV/AIDs, tuberculosis by 2015. The baseline assessment for the health-related Sustainable Development Goals (SDGs) highlights the weaknesses of the Nigerian health system in controlling of endemic diseases [2, 3]. A significant weakness is the scarcity of scientists and health professionals in low and middle-income countries (LMICs) with relevant infectious disease research knowledge and expertise to generate health policy and systems research evidence [4]. A second weakness is the limited use of relevant research evidence for policy and decision making, which essentially hinders well-designed disease control programs from achieving desired goals [5]. There is also the gap in evidence of complex interventions in health to improve knowledge of what works for whom and in what context [6, 7].

As policy-makers and communities increasingly demand better returns on investments in health, HPSR+A has the potential to enable health system interventions to achieve better value for money. However, the current capacity to undertake HPSR+A and teaching is low in developing countries [7]. Health policy and systems research has been defined as “an emerging field that seeks to understand and improve how the societies organize themselves in achieving collective health goals, and how different actors interact in the policy and implementation processes to contribute to health policy outcomes” [8]. It enables the identification of gaps in capacity, barriers to efficient functioning, and effective performance of the health system and methods by which the existing resources can be optimally utilized [9, 10]. Capacity building programs bring on additional resources, i.e. knowledge, skills and experiences in organizational settings [11]. HPSR is typically context-specific, and to apply research evidence to policy, national-level capacity is needed [7]. The success of efforts to build capacity in developing countries will ultimately depend on political will and credibility, adequate financing, and responsive research, capacity, strengthening (RCS) plan that builds on a thorough situational analysis of the resources needed for health research and the inequities and gaps in health care [12].

In order to strengthen health systems using evidence, there is a current need to build the capacity of HPSR+A in LMICs as this encompasses the processes of actual decision-making at all levels of the health system [8]. Capacity building programs are critical for prioritizing health programs in resource-constrained countries where poor health outcomes have been linked with poor health services [11]. There is a rising importance to build capacity in HPSR+A in both the ‘pull and push’ domains of research in Nigeria [13]. This includes capacity to analyse, evaluate, and develop context-specific strategies to strengthen the fight against neglected tropical diseases (NTDs) and malaria. It also encompasses capacity to demand for and use research, so that research knowledge contributes to improvements in health and health equity [13]. Several factors contribute to poor demand for research evidence. First is that there is little appreciation of the value of research and its potential to contribute to policy development. Another critical contributor is that many LMICs do not have an environment or a culture conducive to health research [13]. These environmental factors include governance, socio-political influences and attitude of key stakeholders.

In Nigeria, Universities are central to strengthening and sustaining HPSR+A capacity. They not only produce knowledge through research but are also mandated to teach the next generation of policy-makers, health professionals, and researchers [7]. However, there is limited capacity amongst these groups due to the long-standing culture of not making research a priority and poor funding towards research [1]. The Health Policy Research Group (HPRG), College of Medicine University of Nigeria, Enugu campus (COMUNEC) is currently striving to bring HPSR to the fore in the country by collaborating with policymakers and international partners. They have made some progress, especially in the field of knowledge management for getting research into policy and practice (GRIPP) HPRG comprises public health physicians, medical doctors, epidemiologists, and health economists who are primarily lecturers but use their teaching time to subsidize research [14].

Strengthening the capacity of producers and users of research is arguably a sustainable strategy for developing the field of HPSR+A in Africa than relying on training in high-income countries [10]. As both policy-makers and communities increasingly demand better returns on investments in health, HPSR has the potential to enable health system interventions to achieve better value for money. To reach this potential, producers and users of HPSR evidence need training and local empowerment to be more context useful. World Health Report called for renewed efforts to strengthen health research capacity towards universal health coverage [12], for which capacity building interventions have been identified to bring in new resources (skills, knowledge) in the organization. There is a need for adequate research and analytical capacity in a range of organizations including ministries of health, health policy analysis institutes, think-tanks, academia and civil society in Nigeria. The long-term goal is to strengthen individual and institutional capacity to initiate and lead research activities in disease-endemic countries while developing national and international partnerships. This is timely as there is yet no national policy on the control of NTDs.

This paper provides information on the levels of involvement in HPSR+A (among producers of research evidence) and use of research evidence for decision making (among users of evidence) for the control of endemic diseases in two states in Nigeria. It also highlights potential interventions for improving capacity to undertake and use HPSR+A in policy and decision making.

## Methods

### Study design and area

A quantitative study design was used to collect information from purposively selected respondents who are either researchers/academia (producers of evidence) and policymakers, programme/project managers (users of evidence) in Enugu and Anambra states. A survey questionnaire was administered to 96 respondents in both states.

Enugu and Anambra states are situated in south-east Nigeria. Based on the 2006 census, and an annual growth rate of 2.8%, Enugu state is estimated to have a population of 3.3million people while Anambra state has 4.2million people [15]. The health system in both states is organized in three tiers for service delivery – primary, secondary and tertiary. The State Ministry of Health oversees the affairs of the primary and secondary levels of care. State-owned tertiary hospitals are directly supervised by the State government while their Federal-owned counterparts are directly supervised by the Federal Ministry of Health.

### Study participants

Respondents were purposively selected based on their roles and involvement in endemic disease control in the selected States. They included, i) researchers from universities and research organizations, ii) programme managers for endemic diseases, malaria and maternal and child health, iii) policymakers and senior healthcare managers in State Ministry of Health and affiliated health agencies, iv) data management officers in the Ministry of Health, v) representatives of civil society organizations, and vi) media representatives.

### Data collection

Data was collected data using two different questionnaires for the two categories of respondents (producers and users of research evidence). The questionnaires were designed for this study and reviewed by experts in HPSR+A to ensure contents were valid. They were then were pre-tested for construct validity on similar respondents in Ebonyi state (a neighbouring state) 2 weeks prior to being used to collect data. Feedback from respondents on clarity of questions were used to revise and simplify the questions and options.

The questionnaire for producers of research evidence was used to elicit information on their levels of involvement in HPSR+A (including enablers and constraints), proportion of research time spent in HPSR+A, methods of communication of research findings, level of engagement with policymakers and uptake of research findings for decision making. The questionnaire for users of evidence elicited information on individual and organizational patterns of use of evidence for policy and decision making. It also explored policymakers’ demand for and capacity to initiate research, as well as factors that have enabled or constrained evidence-based decision making. Both questionnaires were also used to collect information on respondents’ personal characteristics such as age, gender, professional cadre and role in organization.

### Data analysis

Univariate analysis was used to summarize categorical variables, while bivariate analysis was undertaken to determine the relationship between respondents’ personal characteristics and their level of involvement in generating and/or using research evidence for decision making. Findings are presented in tables and narratives.

## Results

The personal characteristics of respondents are presented in Table 1. Majority of them were males, 58% of producers and 56.5% of users. Academics (lecturers and professors) accounted for 55.6% of the producers of research evidence, while malaria control was the area of greatest experience for the highest proportion of users of evidence, 26.1%. With respect to job role, half of the producers of evidence were lecturing, and 34.8% of users of evidence reported they were heading departments in their organizations.

**Table 1:** Background information of respondents from both state

Producers of evidence (*N* = Enugu, 23; Anambra,27)				Users of evidence (*N* = Enugu, 21; Anambra, 25)			
Variables	Enugu n(%)	Anambra n(%)	Both n(%)	Variables	Enugu n(%)	Anambra n(%)	Both n(%)
Gender				Gender			
Male	13(56.5)	16(59.3)	29(58.0)	Male	11(52.4)	15(60.0)	26(56.5)
Female	10(43.5)	11(40.7)	21(42.0)	Female	10(47.6)	10(40.0)	20(43.5)
Age group				Age group			
25–40 years	9(39.1)	5(18.5)	14(28.0)	25–40 years	9(42.9)	10(40.0)	19(41.3)
41–50 years	9(39.1)	10(37.0)	19(38.0)	41–50 years	8(38.1)	7(28.0)	15(32.6)
51-60 years	5(21.7)	8(29.6)	13(26.0)	51-60 years	4(19.0)	8(32.0)	12(26.1)
>60 years	–	4(8.0)	4(8.0)	>60 years	–	–	–
Professional cadre				Area of greatest experience			
Lecturer 2	1(4.3)	0(0.0)	1(2.0)				
				Malaria control	8(38.1)	4(16.0)	12(26.1)
Lecturer 1	7(30.4)	2(7.4)	9(18.0)	NTDs	4(19.0)	4(16.0)	8(17.4)
Senior lecturer	3(13.0)	6(22.2)	9(18.0)	HIV/AIDS	2(9.5)	4(16.0)	6(13.0)
Readers	0(0.0)	4(14.8)	10(20.0)	Other communicable diseases	3(14.3)	2(8.0)	5(10.9)
Professor	3(13.0)	3(11.1)	6(12.0)				
Others (e.g. graduate Student)	9(0.0)	12(14.4)	12(44.4)	NCDs	3(14.3)	2(8.0)	5(10.9)
				More than one area	–	8 (32)	8 (17.4)
				Others	1(4.8)	1(4.0)	2(4.3)
Main role in job				Main role in job			
Course coordinator	3(13.0)	4(14.8)	7(14.0)	Departmental head	7(33.3)	9(36.0)	16(34.8)
Departmental head	3(13.0)	6(22.2)	9(18.0)	Divisional head	1(4.8)	1(4.0)	2(4.3)
Dean of faculty	1(4.3)	0(0.0)	1(2.0)	Programme manager	5(23.8)	8(32.0)	13(28.3)
Lecturing	13(56.5)	12(44.4)	25(50.0)	Policymaker	5(23.8)	0(0.0)	5(10.9)
Hospital consultant	2(8.7)	1(3.7)	3(6.0)	Others (e.g. planning officer, statisticians)	3(14.3)	7(28.0)	10(21.7)
Residents doctors	1(4.3)	4(14.8)	5(10.0)				

The rest of the findings are reported separately for producers and users of evidence.

### Findings from producers of evidence

Table 2 shows that 78% of the respondents had ever been involved in HPSR+A, and 52% were currently involved in on-going HPSR+A projects. The percentage of time spent on HPSR+A was 37.1% on the average for all producers of evidence. However, time spent was found to vary significantly across states (*p* = 0.02). The major enablers/motivators to involvement in HPSR+A were personal interest (52%) and mentorship (40%). The major constraint was lack of funds or research grants (62%).

**Table 2:** Level of involvement in HPSR+A among producers of evidence in Enugu and Anambra States

Variables	Enugu (*N* = 23) n(%)	Anambra (*N* = 27) n(%)	Difference □^2^(*p*-value)	Both (*N* = 50) n(%)
Ever been involved in HPSR+A	20(87.0)	19(70.4)	1.991(0.158)	39(78.0)
Involved in any on-going HPSR+A research	16(69.6)	10(37.0)	5.265(0.022)	26(52.0)
Area of current work^a^				
Research	16(69.6)	10(37.0)	4.154(0.042)	26(52.0)
Policy analysis	4(17.4)	5(18.5)	0.011(0.918)	9(18.0)
Policy formulation	1(4.3)	2(7.4)	0.206(0.650)	3(6.0)
Decision making	1(4.3)	3(11.1)	0.772(0.380)	4(8.0)
Research uptake	6(26.1)	2(7.4)	3.224(0.073)	8(16.0)
Implementation research	3(13.0)	3(11.1)	0.044(0.834)	6(12.0)
Operation research	6(26.1)	5(18.5)	0.415(0.520)	11(22.0)
Enabling factors^a^				
Personal interest	16(69.6)	10(37.0)	4.177(0.041)	26(52.0)
Mentorship	13(56.5)	7(25.9)	4.844(0.028)	20(40.0)
Appraisal	8(34.8)	10(37.0)	0.027(0.869)	18(36.0)
Availability of grant	7(30.4)	5(18.5)	0.967(0.508)	12(24.0)
Adequate education resources	5(21.7)	7(25.9)	0.119(0.730)	12(24.0)
Availability of job opportunity	3(13.0)	6(22.2)	0.709(0.400)	9(18.0)
Constraining factors*				
Lack of fund/grants	15(65.2)	16(59.3)	0.187(0.665)	31(62.0)
Lack of interest	5(21.7)	11(40.7)	2.061(0.151)	16(32.0)
Lack of mentorship	5(21.7)	9(33.3)	0.828(0.363)	14(28.0)
Lack of education resources	8(34.8)	5(18.5)	1.708(0.191)	13(26.0)
Limited availability of data	6(26.1)	4(14.8)	0.986(0.321)	10(20.0)
	Mean % (SD)	Mean % (SD)	X^2^(p-value)	Mean % (SD)
% research time spent in HPSR+A	43.9 (±29.2)	29.1 (±18.3)	14.2 (±0.02)	37.1 (±25.6)

^a^multiple responses allowed

With respect to research priority setting, 52% of respondents stated that their research priorities are sometimes informed by the needs of policymakers, and 46% reported that they had undertaken research that was conceived through direct engagement with decision makers.

**Fig. 1:**
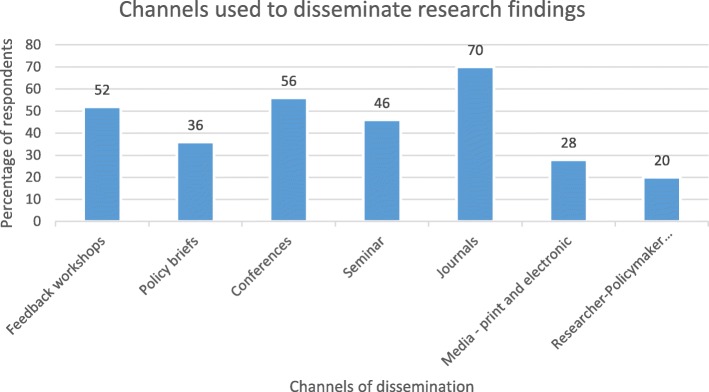
Major channels used by producers to disseminate research findings

Figure 1 shows that the major channels used by producers to disseminate research findings are journal publication (70.0%), conferences (56.0%) and research synthesis feedback workshops (52.0%).

Table 3 shows the association between personal characteristics of producers, their current involvement in an ongoing HSPSR+A project and ever been involved HSPSR+A. There is statistically significant between age category and current involvement in HPSR+A among producers of research.

**Table 3:** Relationship between personal characteristics and involvement in HPSR+A among producers of evidence

Personal characteristics	Ever been involved in HPSR+A	Currently involved in ongoing HSPSR+A project
Gender		
Male	15 (38.5)	12 (46.2)
Female	24 (61.5)	14 (53.8)
□^2^(p-value)	0.911 (0.340)	0.387 (0.370)
Age group		
<25 years	–	
25–40 years	9 (23.1)	11 (42.3)
41 50 years	15 (38.5)	10 (38.5)
51–60 years	11 (28.2)	4 (15.4)
>60 years	4 (10.3)	1 (3.8)
□^2^ (*p*-value)	3.004 (0.391)	7.479 (0.058)
Professional cadre		
Lecturer 2	1 (2.6)	1 (3.8)
Lecturer 1	8 (20.5)	6 (23.1)
Senior lecturer	8 (20.5)	2 (7.7)
Reader	4 (0.3)	2 (7.7)
Professor	6 (15.4)	3 (11.5)
Others	12 (30.8)	12 (46.2)
□^2^ (p-value)	9.670 (0.085)	5.135 (0.400)
Job role		
Course coordinator	4 (15.4)	5 (19.2)
HOD	7 (17.9)	4 (15.4)
Dean	1 (2.6)	0 (0)
Lecturer	21 (53.8)	13 (50.0)
Hospital consultants	2 (5.1)	2 (7.7)
Residents	2 (5.1)	2 (7.7)
□^2^ (p-value)	5.482 (0.360)	2.895 (0.716)

Table 4 shows the relationship between personal characteristics of producers of evidence and areas of involvement in HPSR+A. Significant association was observed between professional cadre and involvement in decision making (p 0.02); and between age category and involvement in policy analysis, policy formulation and decision making (*p* 0.01).

**Table 4:** Relationship between personal characteristics and areas of involvement HPSR+A among producers of evidence

Personal characteristics	Areas of involvement in HPSR+A						
	Research	Policy Analysis	Policy Formulation	Decision Making	Research uptake	Implementation research	Operations research
Gender							
Male	15(45.5)	8(57.1)	2(18.2)	0(0)	8(47.1)	10(52.6)	9(50.0)
Female	18(54.5)	6(42.9)	9(81.9)	9(100)	9(52.2)	9(47.4)	9(50.0)
□^2^(*p*-value)	0.475(0.56)	1.83(0.21)	3.284(0.07)	7.95(0.05)	0.27(0.60)	1.42(0.23)	0.74(0.39)
Age group							
25–40 years	9(27.3)	5(35.7)	1(9.1)	0(0)	6(35.3)	6(31.6)	5(27.8)
41 50 years	13(39.3	3(21.4)	1(9.1)	1(11.1)	3(17.6)	6(31.6)	5(27.8)
51–60 years	8(24.2)	3(21.4)	6(54.5)	5(55.6)	5(29.4)	5(26.3)	6(33.3)
>60 years	3(9.1)	3(21.4)	3(27.3)	3(33.3)	3(17.6)	2(10.5)	2(11.1)
□^2^(p-value)	0.33(0.95)	6.36(0.01)	15.87(0.01)	17.65(0.01)	6.41(0.09)	0.72(0.87)	1.70(0.64)
Academic Cadre							
Lecturer 2	1(3.0)	1(7.1)	1(2.0)	0(0)	1(5.9)	1(5.3)	1(5.6)
Lecturer 1	6(18.2)	4(28.6)	3(27.3)	3(33.3)	4(23.5)	4(21.1)	2(11.1)
Senior lecturer	5(15.2)	2(14.3)	1(9.1)	0(0)	1(5.9)	2(10.5)	5(27.8)
Reader	4(12.1)	1(7.1)	2(18.2)	2(22.2)	1(5.9)	2(10.5)	16.7)
Professor	5(15.2)	2(14.3)	3(27.3)	3(33.3)	3(17.6)	4(21.1)	2(11.1)
Others	12(36.4)	4(11.1)	2(18.2)	1(11.1)	7(41.2)	6(31.6)	5(27.8)
□^2^(p-value)	4.55(0.473)	4.87(0.43)	8.05(0.15)	13.06(0.02)	5.31(0.38)	5.87(0.32)	8.03(0.16)
Job role							
Coordinator	5(15.2)	3(21.4)	2(18.2)	1(11.1)	3(17.6)	4(21.1)	5(27.8)
HOD	7(21.2)	2(14.3)	3(27.3)	5(55.6)	5(29.4)	2(10.5)	2(11.1)
Dean	0(0)	0(0)	0(0)	0(0)	0(0)	1(5.3)	0(0)
Lecturer	18(54.5)	8(57.1)	4(36.4)	3(33.3)	8(47.1)	11(57.9)	10(55.6)
Hospital Cons	2(6.1)	0(0)	1(6.1)	0(0)	1(5.9)	1(5.3)	0(0)
Residents	1(3.0)	1(7.1)	1(9.1)	0(0)	0(0)	0(0)	1(5.6)
□^2^(p-value)	7.71(0.17)	2.83(0.73)	1.89(0.86)	11.25(0.06)	5.24(0.39)	7.15(0.21)	7.534(0.18)

Relationship between personal characteristics of producers of evidence and proportion of research time spent on HPSR+A is presented in Table 5. Significant association was observed between professional cadre and percentage of research time spent on HPSR+A (p, 0.04). Lower cadres of professionals appeared to spend more time in HPSR+A.

**Table 5:** Relationship between personal characteristics and time spent on HPSR+A among producers of evidence

Personal characteristics	Time spent on HPSR+A as a proportion of research work					
	</=20%	21–40%	41–60%	61–80%	> 80%	□^2^(*p*-value)
Gender						
Male	8(66.7)	6(75.0)	2(25.0)	1(33.3)	1(50.0)	5.439(0.245)
Female	4(33.3)	2(25.0)	6(75.0)	2(66.7)	1(50.0)	
Age group						
25–40 years	3(25.0)	1(12.5)	6(75.0)	2(66.7)	0(0)	
41 50 years	5(41.7)	3(37.5)	1(12.5)	0(0)	2(100.0)	15.525(0.214)
51–60 years	2(16.7	3(37.5)	1(12.5)	1(33.3)	0(0)	
>60 years	2(16.7)	1(12.5)	0(0)	0(0)	0(0)	
Professional cadre						
Lecturer 2	0(0)	0(0)	0(0)	1(33.3)	0(0)	
Lecturer 1	0(0)	2(25.0)	1(12.5)	2(66.7)	1(50.0)	
Senior lecturer	3(25.0)	1(12.5)	2(25.0)	0(0)	0(0)	32.271(0.040)
Reader	1(8.3)	2(25.0)	0(0)	0(0)	0(0)	
Professor	5(41.7)	1(12.5)	0(0)	0(0)	0(0)	
Others	3(25.0)	2(25.0)	5(62.5)	0(0)	1(50.0)	
Job role						
Course coordinator	2(16.7)	1(12.5)	1(12.5)	1(33.3)	1(50.0)	
Departmental head	1(8.3)	1(12.5)	1(12.5)	0(0)	0(0)	
Dean	1(8.3)	0(0)	0(0)	0(0)	0(0)	
Lecturer	6(50.0)	5(62.5)	5(62.5)	2(66.7)	1(50.0)	
Hospital consultants	1(8.3)	0(0)	0(0)	0(0)	0(0)	6.569(0.998)
Residents	1(8.3)	1(12.5)	1(12.5)	0(0)	0(0)	

### Findings from users of evidence

Over 95% of users of evidence reported that they were aware that research evidence could be used for decision making. Table 6 shows that 82.6% of respondents in both States reported they had ever used evidence for decision making. Significant variation was seen between both states with 95.2% in Enugu and 72% in Anambra. The most common type of evidence used were findings from research and surveys (60.5%), and this was also seen to vary between the States, with 77.8% of users in Anambra and 45% in Enugu state. Use of HPSR evidence for decision making was reported by 56.5% of respondents. The main reasons for non-use of evidence from HPSR were lack of awareness (32.6%) and limited decision making autonomy (21.7%).

**Table 6:** Pattern of use of research evidence for policy/decision making among users of evidence

Variables	Enugu (*N* = 21) n(%)	Anambra (*N* = 25) n(%)	Difference □^2^(p-value)	Both (*N* = 46) n(%)
Ever used evidence	20(95.2)	18(72.0)	**4.290(0.038)**	38(82.6)
Currently uses evidence	19(90.5)	19(76.0)	1.665(0.197)	38(82.6)
Type of evidence used				
Data from Federal Ministry of Health (including HMIS data)	7(35.0)	3(16.7)		10(26.4)
Research evidence/Surveys	9(45.0)	14(77.8)	12.516(0.051)	23(60.5)
Program Reports	0(0.0)	1(5.6)		1(2.6)
Secondary data	1(5.0)	0(0.0)		1(2.6)
Data Quality Assessment	2(10.0)	0(0.0)		2(5.3)
Situation analysis	1(5.0)	0(0.0)		1(2.6)
Ever used evidence from HPSR	12(57.1)	14(56.0)	0.006(0.938)	26(56.5)
Reason for not using evidence from HPSR^a^				
Not aware of the field of HPSR+A	6(28.6)	9(36.0)	0.287(0.592)	15(32.6)
Lack of decision making autonomy	5(23.8)	5(20.0)	0.097(0.755)	10(21.7)
Others	2(9.5)	1(4.0)	0.571(0.585)	3(6.5)

^a^Multiple response allowed; HMIS refers to Health management information system

Percentage of times decisions were made based on research evidence are summarized in Table 7. On the average, respondents reported that any type of evidence was used to make decisions 54.5% of times while HPSR evidence was used 41.4% of times.

**Table 7:** Proportion of times users of evidence made decisions based on research evidence

Variables	Enugu (*N* = 21) Mean %	Anambra (*N* = 25) Mean %	Difference □^2^(p-value)	Both (N = 46) Mean %
Any type of evidence used in decision making	50.26(19.62)	58.550(20.68)	2.237(0.071)	54.51(20.35)
Evidence from HPSR used in decision making	48.75(18.72)	34.615(15.06)	3.931(0.052)	41.40(18.06)
Evidence used in programme design & implementation specifically	58.235(16.00)	50.882(21.08)	2.155(0.055)	54.56(18.80)

**Fig. 2:**
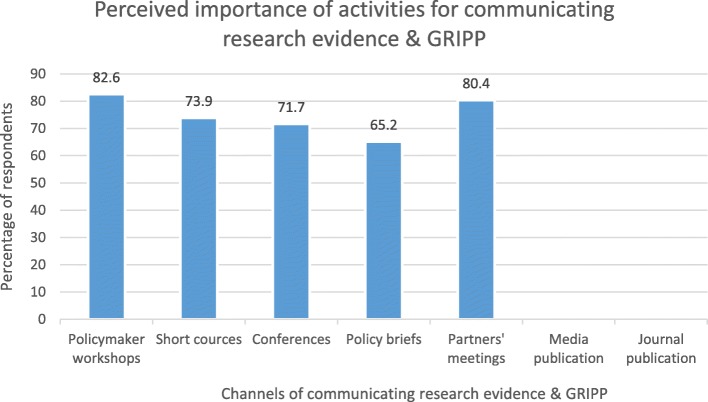
Perceptions of importance of specific GRIPP activities for communicating research evidence to policy and decision makers

Figure 2 highlights perceptions of importance of specific GRIPP activities for communicating research evidence to policy and decision makers. Users of evidence perceived policymaker workshops (82.6%), partners’ meetings (80.4%), short courses (73.9%) and conferences (71.7%) as importance channels for communication research evidence to policymakers. Synthesis of research evidence as policy briefs was also considered an important communication channel by 65.2% of users of evidence.

Table 8 shows that there is no significant association between respondents’ personal characteristics and their use of evidence for policy and decision making.

**Table 8:** Relationship between personal characteristics and use of research evidence for policy and decision making

Background information	Ever used research evidence n(%)	Currently uses research evidence n(%)	Ever used HPSR evidence n(%)
Gender			
Male	15(39.5)	15(39.5)	11(42.3)
Female	23(60.5)	23(60.5)	15(57.7)
□^2^(*p*-value)	1.426(0.267)	1.426(0.232)	0.033(1.000)
Age group			
25–40 years	17(44.7)	17(44.7)	9(34.6)
41–50 years	12(31.6)	11(28.9)	9(34.6)
51-60 years	9(23.7)	10(26.3)	8(30.8)
□^2^(p-value)	1.178(0.555)	1.526(0.466)	1.224(0.542)
Main role in job			
Departmental head	11(28.9)	11(28.9)	7(26.9)
Divisional head	2(5.3)	2(5.3)	2(7.7)
Programme manager	12(31.6)	12(31.6)	7(26.9)
Policymaker	5(13.2)	5(13.2)	5(19.2)
Others	8(21.1)	8(21.1)	5(19.2)
□^2^(p-value)	4.511(0341)	4.511(0.341)	6.658(0.155)

## Discussion

The higher proportion of producers of research evidence involved in HPSR+A in Enugu state could be explained by the influence of Health Policy Research Group which is a foremost organization known for generating research evidence in health policy and systems analysis, as well as training potential HPSR+A researchers. Enablers for involvement in HPSR+A include personal interest, mentorship, promotion and availability of research grants. On the flip-side, respondents reported that lack of funds, lack of interest, lack of mentorship and lack of educational resources were major constraints to getting involved in HPSR+A. These results are in concordance with other studies that have highlighted funding, mentorship and opportunities for training as key drivers for building capacity of researchers in the field of HPSR+A [7, 9, 12–14].

Majority of the users of evidence (decision/policymakers) in both states were aware that research evidence should inform policy decisions, and a few reported they had in the past requested for research evidence or initiated research for policy/strategy development and program implementation/review. This could be attributed to increasing global attention and support for evidence-informed decision making in health. The proportion of users who reported actually using research evidence in decision making was not as robust, and their reasons were mainly lack of awareness of HPSR evidence and lack of autonomy in decision making. The former reason is irrespective of the fact that considerable numbers of producers of research evidence in both States reported that their research is informed by health policy and program priorities of users of evidence. Poor access to research evidence and limited decision space have been previously reported as constraints to evidence-based decision making [16, 17].

There seemed to be a clear channel for gathering evidence for decision making in both States. Whereas producer of research evidence preferred journal publications, conferences and feedback workshops for dissemination of research findings, users of evidence appeared to value policymaker workshops, partners’ meetings and short courses as important channels for communicating/receiving research evidence. The preference for journal publication among producers of evidence is due to high visibility and academic staff appraisal requirements which are both critical for career progression of university lecturers. The observed mismatch in communication of research evidence has been underscored as a major constraint to GRIPP [14]. Hence communication of research evidence should occupy a major part of interventions for improving capacity in HPSR+A in LMICs.

Finally, although, users of evidence in both States perceived research dissemination and GRIPP activities to be very important, their capacity to participate in these activities was poor, and has been linked to low demand for high-quality research outputs [18]. This underlines the importance of bridging the gap between research and policy making. Decision makers need capacity building in order to, i) better understand the field of HPSR+A, ii) be able to initiate and commission health systems research that are relevant for policy and decision making, iii) be able to source for, synthesize and use research evidence for policy making. Similarly, because HPSR+A is a relatively new field, there is a need to continue building a critical mass of researchers that will be able to undertake such studies and have the skills for GRIPP.

### Strengths and limitations of the study

The study elicited information from a diverse group of respondents who represent the two vital categories of actors needed for getting research evidence into policy and practice, and uses quantitative research method which has not been extensively applied in evaluating capacity needs for HPSR+A. Notwithstanding that it focuses on two Nigerian states, findings could be applied in settings with similar contexts. The application of quantitative method alone is a major limitation because it does not allow in-depth exploration of the subject matter. However, other authors have examined HPSR+A capacity needs using qualitative research methods. This paper also highlights a knowledge gap of respondents’ views of what could be done to ensure optimal integration of research evidence into policy and practice in the control of endemic diseases. This could form the basis for further study.

## Conclusion

There are gaps in capacity to produce and use evidence for decision making in control of endemic diseases in Nigeria. Involvement of researchers in HPSR+A is constrained by lack of funding, limited numbers of mentors and inadequate training opportunities. Poor uptake of research evidence in policymaking is hindered by poor access to research evidence and lack of autonomy in decision making. There is the need to invest in capacity building activities in order to develop a critical mass of users and producers of evidence in HPSR+A for better control of endemic diseases.

## Data Availability

The datasets used and/or analysed during the current study are available from the corresponding author on reasonable request.

## Abbreviations

COMUNEC: College of Medicine University of Nigeria, Enugu Campus
GRIPP: Getting Research into Policy and Practice
HPRG: Health Policy Research Group
HPSR: Health Policy and Systems Research
HPSR+A: Health Policy and Systems Research and Analysis
LMICs: Low and Middle-Income Countries
MDG: Millennium Development Goals
NCD: Non-communicable diseases
NTDs: Neglected Tropical Diseases
RCS: Research Capacity Strengthening
SDGs: Sustainable Development Goals
